# Systematic Surveillance of an Emerging Picornavirus among Cattle and Sheep in China

**DOI:** 10.1128/spectrum.05040-22

**Published:** 2023-05-10

**Authors:** Chengyuan Ji, Yao Zhang, Yiqiu Feng, Xinqin Zhang, Jiale Ma, Zihao Pan, Atsushi Kawaguchi, Huochun Yao

**Affiliations:** a Ministry of Education Joint International Research Laboratory of Animal Health and Food Safety, College of Veterinary Medicine, Nanjing Agricultural University, Nanjing, China; b Department of Infection Biology, Faculty of Medicine and Graduate School of Comprehensive Human Sciences, University of Tsukuba, Tsukuba, Japan; U.S. Food and Drug Administration

**Keywords:** emerging, picornavirus, boosepivirus, coinfection, spillover

## Abstract

Emerging viruses are a constant threat to human and animal health. Boosepivirus is a novel picornavirus considered a gastrointestinal pathogen and has broken out in recent years. In 2020, we identified a strain of boosepivirus NX20-1 from Chinese calf feces and performed genetic characterization and evolutionary analysis. NX20-1 was closely related to the Japanese strain Bo-12-38/2009/JPN and belonged to Boosepivirus B. We found that 64 of 603 samples (10.6%) from 20 different provinces across the country were positive for boosepivirus by reverse transcription (RT)-PCR. Further, coinfection with other diarrheal pathogens was also present in 35 of these positive samples. Importantly, we found the prevalence of boosepivirus in sheep as well, indicating that Boosepivirus can infect different domestic animals. Our data suggest that boosepivirus is a potential diarrheal pathogen, but the pathogenicity and the mechanism of pathogenesis need further study.

**IMPORTANCE** We identified a novel picornavirus, boosepivirus, for the first time in China. Genetic evolutionary analysis revealed that NX20-1 strain was closely related to the Japanese strain Bo-12-38/2009/JPN and belonged to Boosepivirus B. In addition, we found that the virus was prevalent in China with an overall positivity rate of 10.6% (64 of 603 samples), and there was significant coinfection with other pathogens. Importantly, we found the prevalence of boosepivirus in sheep as well, suggesting that boosepivirus has a risk of spillover and can be transmitted across species.

## INTRODUCTION

Most members of the *Picornaviridae* family comprise a significant burden among humans and animals, causing diseases such as gastroenteritis, hepatitis, and other diseases. This family currently consists of 158 species divided into 68 genera (as of March 2022), and with the widespread use of next-generation sequencing (NGS) technology, the family members will continue to expand. Recently, several novel picornaviruses were discovered from different hosts, including Macaca mulatta, clownfish, penguins, and bats (1–4). Boosepivirus (BooV) was a novel genus of the family *Picornaviridae* proposed in 2020, and the name was derived from bovine, ovine sapelo-entero-like picornavirus (https://www.picornaviridae.com/ensavirinae/boosepivirus/boosepivirus.htm). Based on their common genome structure and high amino acid (aa) identity (aa >70% in the polyprotein, aa >60% in P1, and >65% aa identity in 2C + 3CD), the genus *Boosepivirus* is classified into three genera: Boosepivirus A, Boosepivirus B, and Boosepivirus C.

BooV was first identified in feces from cattle with diarrhea in 2009 in Hokkaido Prefecture, Japan, and nearly complete sequences of the virus were determined by the metagenomics approach (5). Further testing of fecal samples from cattle with diarrhea in Hokkaido was subsequently performed, and 23.0% (20 of 87) of them were positive for BooV, indicating that BooV is widespread among cattle in Hokkaido. BooV was isolated from cattle with diarrhea not only in Japan but also in the United States, and further pathogenetic investigations revealed that it has been widely prevalent in the region (5, 6). These reports suggest that BooV is closely associated with diarrhea in cattle.

Diarrhea remains a major cause of productivity and economic losses for cattle producers worldwide. In addition to the major enteric pathogens such as bovine rotavirus (BRV), bovine coronavirus (BCoV), bovine viral diarrhea virus (BVDV), Salmonella enterica, and Escherichia coli, a variety of emerging enteric pathogens, including bovine enterovirus (BEV) (7–9), bovine astrovirus (BAstV) (10–12), bovine kobuvirus (BKV) (13–16), bovine torovirus (BToV) (17–19), and bovine norovirus (BNoV) (20, 21), are involved in the development of this disease.

In clinical diagnostic diarrhea panel samples, there are often negative results for known diarrheal pathogens, suggesting the possibility of unknown pathogens. The identification of novel pathogens is particularly important to deepen the knowledge of disease etiology. In this study, we report on an occurrence of severe diarrhea in a herd of calves in Ningxia, China, and BooV was identified as the causative pathogen by NGS. Further pathogen testing revealed that BooV is widely prevalent in cattle in different regions of the country, while coinfected with other pathogens. Notably, BooV infection was also present in sheep herds, suggesting that BooV can infect different domestic animals.

## RESULTS

### Identification of a novel picornavirus by metagenomic sequencing.

In December 2020, two watery, creamy yellow calf feces samples were delivered from a dairy farm in Ningxia Province, China, within our pathogen surveillance system. These two calves were about 2 weeks old, and we performed routine pathogen testing, including BRV, BCoV, BVDV, S. enterica, and E. coli. One sample was strongly positive for BRV with a Ct value of 22.4, but the other sample did not contain any of the common pathogens. Illumina sequencing was used to analyze stool samples from cattle diarrhea cases, which resulted in a total of 36,262,541 reads. After the removal of host factors, a total of 55,607 reads were obtained, of which 3,899 were identified as viruses. Among these viral reads, BooV was discovered and assembled, resulting in a full-length BooV sequence of 7,616 nucleotides. We named the BooV strain NX20-1 and found a close association with the newly discovered Japanese strain of bovine picornavirus BooV (Bo-12-38/2009/JPN). We then performed reverse transcription (RT)-PCR on another sample that had been positive for BRV, and the result showed a strong positive result for BooV as well.

### Genome characterization and phylogenetic analysis.

The length of NX20-1 viral genome (GenBank accession no. OP554215) is 7,617 nt, and NX20-1 has a large open reading frame (ORF) (7,023 nt; 2,341 aa). NX20-1 had the highest identity in complete genome sequence with Boosepivirus B Bo-12-38/2009/JPN strain at 98.2%, followed by Bo-12-11/2009/JPN at 97.4% and other Boosepivirus B strains at 84.1% to 86.6%. However, the identity of NX20-1 with Boosepivirus A and C was low, ranging from 57.8% to 58.9% (Table 1). Similar to the results of the complete genome, P1 protein of NX20-1 showed high identity with Bo-12-38/2009/JPN compared to other Boosepivirus B strains.

**TABLE 1:** Sequence identities of bovine boosepivirus strain NX20-1 with other reference strains^*a*^

Accession	Strain	Host	Complete genome	Polyprotein	P1	2C + 3CD	Genus
LC006971	Bo-11-39/2009/JPN	Bovine	57.8	54.0	56.4	61.0	Boosepivirus A
LC036581	Bo-12-11/2009/JPN	Bovine	97.4	99.4	100	99.0	Boosepivirus B
LC036582	Bo-12-38/2009/JPN	Bovine	98.2	99.7	100	99.5	Boosepivirus B
LR216006	Ovine picornavirus	Ovine	58.9	53.9	58.9	57.8	Boosepivirus C
LR216007	Ovine picornavirus	Ovine	58.8	53.9	58.9	57.8	Boosepivirus C
MZ052226	21-0305	Bovine	86.6	98.5	99.3	98.5	Boosepivirus B
OK247513	IL41203-19	Bovine	84.8	95.8	91.2	98.9	Boosepivirus B
ON168931	BoP6/2021/CHN	Bovine	84.6	95.6	91.2	98.3	Boosepivirus B
OP062271	BoP9/2021/CHN	Bovine	84.1	95.6	91.2	98.8	Boosepivirus B

a Except for the percentage identity of the complete genome at the nucleotide level, all other percentages of identity are at the protein amino acid level. The values represent the percentage of identity.

**FIG 1:**
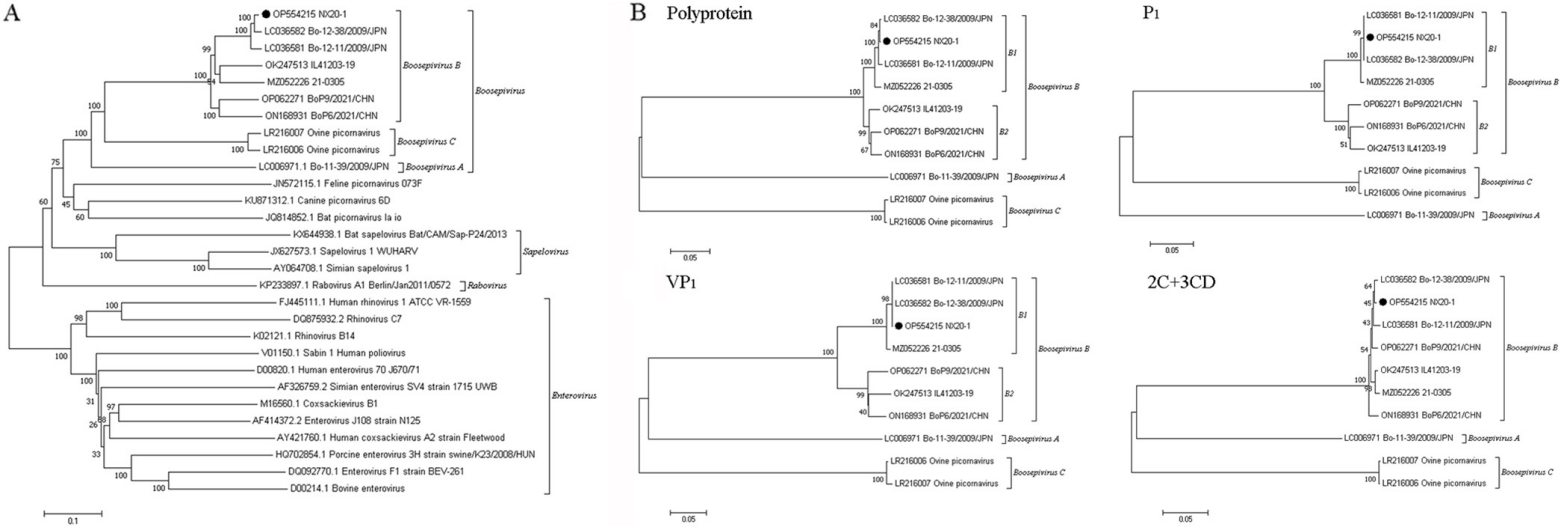
Phylogenetic analyses based on the complete genome and amino acid sequences of polyprotein, P1, VP1, and 2C + 3CD. The phylogenetic trees were built using the neighbor-joining method with 1,000 bootstrap replicates in MEGA7 software. Black circles represent boosepivirus (BooV) NX20-1 strains identified in this study.

Phylogenetic analysis based on the nucleotide sequence of viral genome and the amino acid sequence of polyprotein, P1, and VP1 also indicates that NX20-1 is closely related to Bo-12-38/2009/JPN strain of the Boosepivirus B species (Fig. 1). Meanwhile, Boosepivirus B can be further divided into two subtypes: B1 and B2, of which the NX20-1 and Bo-12-38/2009/JPN strains belong to subtype B1. Recombination analysis showed that this strain does not recombine with other BooVs (data not shown).

### Epidemiological investigation of BooV infection in China.

**FIG 2:**
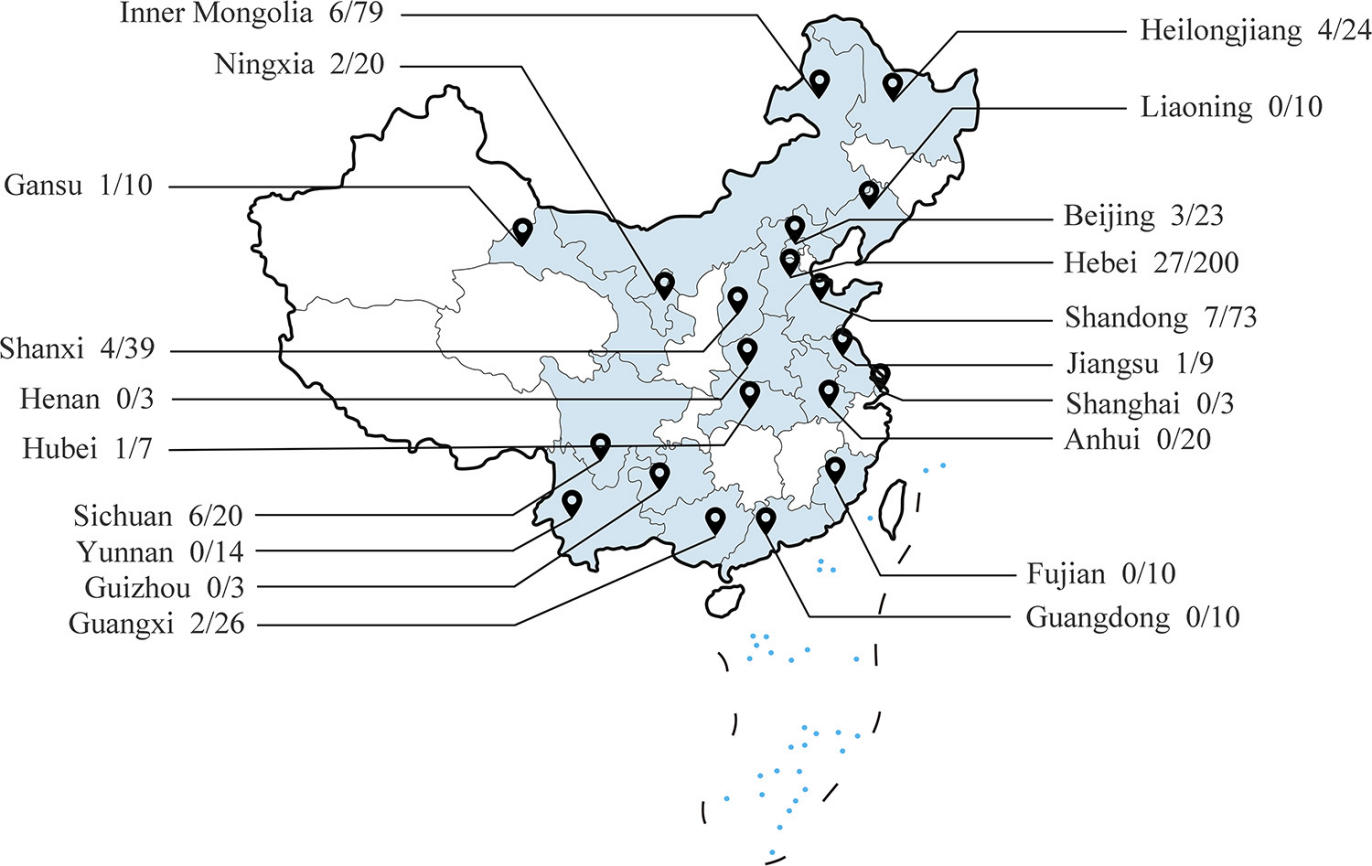
Locations of BooV sample collection sites. Cattle samples were collected in 20 provinces of China.

**FIG 3:**
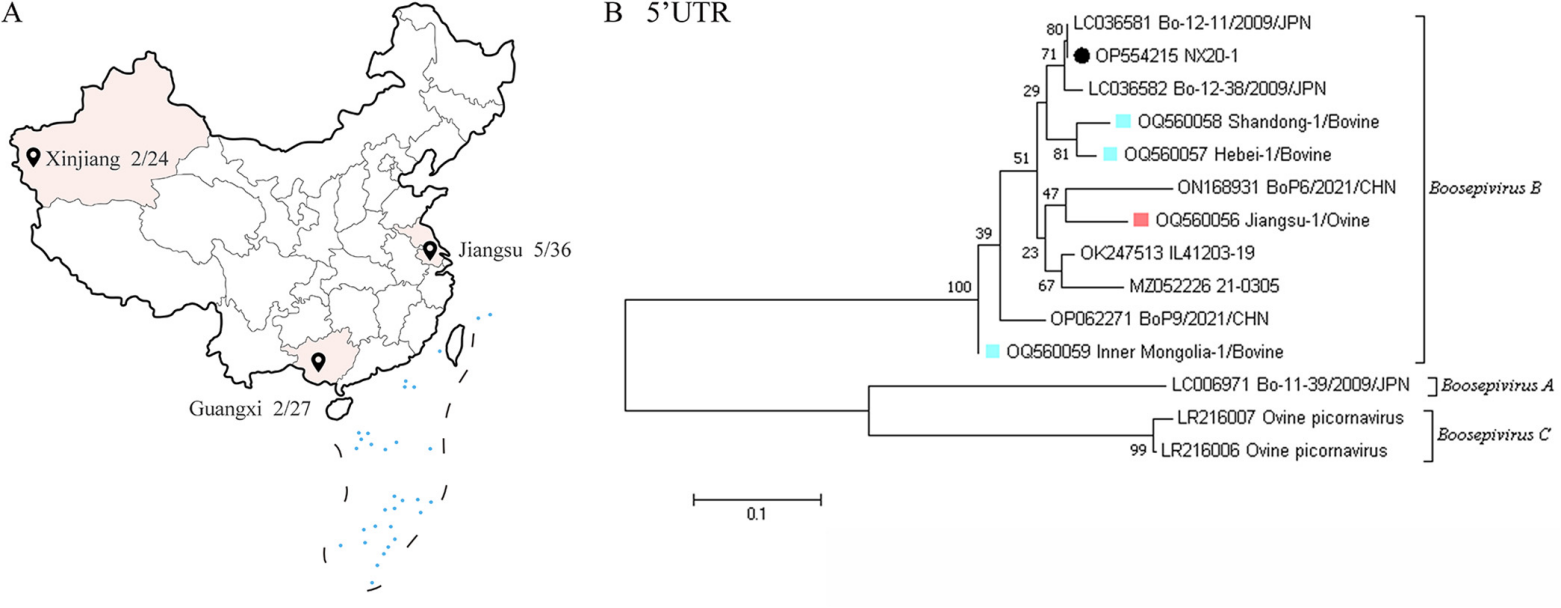
Locations of BooV sample collection sites and phylogenetic analysis. (A) Three provinces of China where ovine samples were collected. (B) Phylogenetic analysis of 5′-untranslated region (UTR) in clinically tested positive samples and Kimura-2 model of nucleotide substations was used. The black circle represents the BooV NX20-1 strain identified in this study. Blue squares represent positive samples from cattle, and the pink square represents a positive sample from sheep. UTR, untranslated region.

To evaluate the frequency of BooV infection, a total of 603 samples were collected from 75 different farms in 20 provinces of China and investigated by RT-PCR. A total of 64 samples (64 of 603) tested positive and 27 farms were positive for BooV. From Fig. 2, BooV was detected in 12 provinces (12 of 20), and the prevalence appears to be higher in the northern provinces of China. As a potential diarrhea pathogen, we tested these 64 positive samples for other diarrhea-related viruses (BRV, BCoV, and BVDV). As shown in Table 2, coinfections were present in more than half of the samples, with coinfection with BRV being the most frequent. As an emerging picornavirus, we were interested in whether BooV has a risk of spillover. We collected a total of 87 sheep feces samples from three distantly located provinces: Guangxi (*n* = 27), Jiangsu (*n* = 36), and Xinjiang (*n* = 24), for testing and found 9 positive feces, and boosepivirus was detected in all three provinces (Fig. 3A). We performed a phylogenetic analysis based on partial 5′-untranslated region (UTR) sequences and found that Boosepivirus B was the main prevalent strain in both cattle and sheep populations (Fig. 3B). These results suggest that BooV has become widespread in cattle and sheep in China with a risk of spillover.

**TABLE 2:** Percentage of 64 BooV coinfection combination^*a*^

Patterns of coinfection	Sample no.
BooV + BRV	16
BooV + BCoV	12
BooV + BVDV	7
BooV + BRV + BcoV	3
BooV + BRV + BVDV	2
BooV + BcoV + BVDV	2
BooV + BRV + BcoV + BVDV	1

a BCoV, bovine coronavirus; BooV, boosepivirus; BRV, bovine rotavirus; BVDV, bovine viral diarrhea virus.

## DISCUSSION

The unprecedented coverage provided by NGS technology facilitates the extraction and inference of important epidemiological information about clinical samples without viral isolation to determine total genomic information within samples, enabling the development of more effective prevention strategies and antiviral therapies (8). Recently, NGS has become well established for the evaluation of virome, including novel viruses in clinical samples (9–11). In this case, we used this technique to identify a novel picornavirus, boosepivirus, from calf diarrhea feces and hypothesized that this might be the pathogen responsible for calf diarrhea.

Calf diarrhea continues to be a problematic issue on farms; more than 50% of preweaning calf mortality is associated with diarrhea, with the majority of cases occurring in calves less than 1 month of age (12). Calf diarrhea caused by infectious agents is associated with significant economic losses in the cattle industry due to calf mortality and growth retardation. Although a single major pathogen may be the cause in some cases, coinfection is frequently observed in calves with diarrhea (13–15). In our initial diagnosis, one of the stool samples was a coinfection of BRV with BooV. BooV is a potential cause of diarrhea, so we were interested in its coinfection with other diarrhea viruses. We selected three significant enteric disease viruses (BRV, BCoV, and BVDV) for testing and showed coinfection in more than half (*n* = 35) of all 64 BooV-positive samples. Previous studies have also demonstrated the widespread coinfection of BooV with other pathogens (6, 16). Unfortunately, we have not been able to successfully isolate the virus to explore its pathogenicity. However, through our pathogenetic investigations, we believe that BooV is a potential diarrheal pathogen and is often coinfected with other pathogens.

Picornaviruses are known to exhibit highly variable tissue tropism and the ability to easily cross interspecies barriers, leading to very diverse disease syndromes, including neurological, respiratory, enteric, and systemic (17, 18). The picornavirus has a wide range of hosts, such as the genus Enterovirus infect hosts ranging from humans and simian to cattle, goats, pigs, and even beyond (19–24). The genus Boosepivirus contains three proposed genera: Boosepivirus A, Boosepivirus B, and Boosepivirus C. Our results suggest that Boosepivirus B is the predominant strain currently prevalent in the Chinese cattle population. Even though Boosepivirus was first identified in cattle, given the strong cross-species transmission of picornavirus, we also collected fecal samples from sheep in three different provinces. We found that Boosepivirus B could infect not only cattle but also sheep, with the possibility of interspecies transmission, indicates that Boosepivirus can infect different domestic animals.

### Conclusions.

This study reports for the first time the detection and genetic characterization of BooV strain NX20-1 of genus Boosepivirus B in calf feces in China and found that BooV is frequently coinfected with other diarrhea viruses. We conclude that BooV has become widespread in Chinese livestock animals and that Boosepivirus B is the predominant endemic strain. We recommend ongoing BooV surveillance in humans and multiple animals to understand and control the circulation of this potentially pathogenic virus.

## MATERIALS AND METHODS

### Clinical sample collection.

In this study, 603 clinical samples of cattle diarrhea, including feces, anal swabs, or intestinal tissues, were obtained from 75 farms in 21 different regions of China from November 2019 to January 2022. An additional 87 sheep fecal samples were obtained from Jiangsu, Guangxi, and Xinjiang. We collected samples after being granted permission by the relevant farm owners.

### RNA extraction of viruses.

Each sample was homogenized in the 5-fold volume of phosphate-buffered saline (PBS) (pH 7.2). The viral RNA was extracted using the Vazyme FastPure viral DNA/RNA mini kit (Nanjing, China) from 200 μL of clinical samples. All extracted RNA or DNA was dissolved in 50 μL of nuclease-free water and stored in a refrigerator at −80°C until used for the assay.

### Library construction and Illumina HiSeq sequencing.

Metagenomic shotgun sequencing libraries were constructed and sequenced at Shanghai Biozeron Biological Technology Co., Ltd. Briefly, for each sample, 1 μg of genomic DNA was sheared by a Covaris S220 focused ultrasonicator (Woburn, MA, USA), and sequencing libraries were prepared with a fragment length of approximately 450 bp. All samples were sequenced in the Illumina HiSeq X instrument with pair-end 150 bp (PE150) mode. Raw sequence reads underwent quality trimming using (https://github.com/usadellab/Trimmomatic) to remove adaptor contaminants and low-quality reads (25). The reads removing host-genome contaminations and low-quality data were called clean reads and used for further analysis.

### Genome and phylogenetic analysis.

The complete gene sequences of boosepivirus obtained in this study have been uploaded to GenBank with the accession number OP554215. Subsequently, all arrangements were further aligned with MegAlign (Lasergene) using the ClustalW alignment method. Phylogenetic trees were built using the neighbor-joining method with 1,000 bootstrap replicates in MEGA7 software. Genomic sequences of BooV NX20-1 and other BooV strains were analyzed using RDP4 to detect possible recombination events. Seven methods were applied, including RDP, GENECONV, Chimaera, MaxChi, 3Seq, SiScan, and BootScan. At least six methods with *P* values less than 0.05 were required for sequences to be considered recombinant and subject to further analysis. The parameters were set with a window size of 500 bp and a step size of 50 bp (25).

### Retrospective research.

To investigate the prevalence of BooV in cattle farms, we conducted national surveillance of BooV with reference to RT-PCR as described previously (5). They designed a pair of universal primers based on the nucleotide sequence of the 5′-UTR of BooV (forward, 5′-CTTTTTCCCCCTCTTGYAAC-3′; and reverse, 5′-TTAGCCGCATTCAGGGKCCTGGAG-3′), which resulted in a PCR product of approximately 338 bp. The amplification of fragments was carried out under the following conditions: 3 min at 95°C, followed by 30 cycles of 30 s at 95°C and 30 s at 55°C, and 30 s at 72°C, with a final extension at 72°C for 5 min. The RT-PCR product was electrophoresed and purified on a 1.5% agarose gel. To address the possibility of coinfections of BooV with other viruses, we tested BRV, BCoV, and BVDV with the primers shown in Table 3. To confirm the risk of spillover of BooV, we collected 87 sheep samples from three different regions for the test.

**TABLE 3:** Primers used in the study^*a*^

Primer	Sequence (5′ to 3′)	Region	Accession no.
BVDV-1F	GACTGTTACGAATACAGCCTGATAGG	5′-UTR	NC001461
BVDV1-R	GTGCCATGTACAGCAGAGATTTTT		
BVDV1-Probe	TGCTGCAGAGGCCCACTGTATTGCTAC		
BRV-F	GAACAGATCACAACCAGCTCATG	VP6	MN047454
BRV-R	CGAATCCAGCGACCTGAATTTC		
BRV-Probe	CCCGCATTGAGCCACATCGTACCC		
BCoV-F	GGACCCAAGTAGCGATGAGG	N	ON142320
BcoV-R	GCAGACCTTCCTGAGCCTTC		
BcoV-Probe	CCGACTAGGTTTCCGCCTGGCACG		

a F, forward; R, reverse; UTR, untranslated region.

### Ethics approval and consent to participate.

All clinical samples used in this study were diagnostic samples submitted by the clients. Therefore, no animal-handling activities related to ethical issues were involved in this study.

### Availability of data and materials.

All relevant experimental data are presented in the article. The detailed information on the viral strains that were included in the current study have been deposited in the NCBI database and are already available to the public.
